# Health Care Use and Physical, Psychological, Cognitive, and Social Frailty in Community-Living Adults 45-85

**DOI:** 10.1093/geroni/igaf122.1089

**Published:** 2025-12-31

**Authors:** Lauren Griffith, Graciela Muniz-Terrera, Edwin van den Heuvel, Jayati Khattar, David Hogan, Megan E O’Connell, Mélanie Levasseur, Parminder Raina

**Affiliations:** McMaster University, Hamilton, Ontario, Canada; Ohio University, Athens, Ohio, United States; Eindhoven University of Technology, Eindhoven, Noord-Brabant, Netherlands; University of Toronto, Toronto, Ontario, Canada; University of Calgary, Calgary, Alberta, Canada; University of Saskatchewan, Saskatoon, Saskatchewan, Canada; Université de Sherbrooke, Sherbrooke, Quebec, Canada; McMaster University, Hamilton, Ontario, Canada

## Abstract

This study examines associations between physical, psychological, social, and cognitive frailty domains with health care utilization (HCU) and the potential moderating effect of the last three domains on the association between physical frailty and HCU. A 127-item Frailty Index (FI) developed for the Canadian Longitudinal Study on Aging comprehensive cohort (n = 30,097) was used to create physical, psychological, cognitive, and social domain-specific FIs. Each FI was divided into quintiles with the highest 20% representing the frailest. Logistic regression was used to estimate unadjusted and adjusted (covariates: sex, age, income, smoking, physical activity, nutrition, and participation restriction) ORs (aORs) for frailty domains and HCU (formal/informal care, family physician visits, hospitalizations) and interactions between physical frailty and the other frailty domains. Physical frailty was associated with the highest HCU ORs in unadjusted (1.53 to 2.38) and adjusted (1.28 to 1.78) analyses, with the largest aOR for formal care (1.78, 95% CI 1.66, 1.91). For all HCU except formal care, the upper CI limits for social frailty were < 1, indicating those with higher levels of social frailty were less likely to use these services. Interactions between physical frailty and the other frailty domains were significant for only formal and informal care, with the aOR magnitudes for the other domains increasing with the level of physical frailty. Our data suggests that the drivers of HCU are multifactorial and the need to consider both frailty beyond physical characteristics and the complex relationships between frailty domains and HCU when assessing the outcomes of frailty interventions.

